# Supplementary figures and images for: The role of B12 deficiency and methionine synthase in methionine-dependent cancer cells
Source: Cancer Metab. 2025 Jul 2;13:34. doi: 10.1186/s40170-025-00405-2 (PMC12220533; doi:10.1186/s40170-025-00405-2)

# Supplementary Figure 1

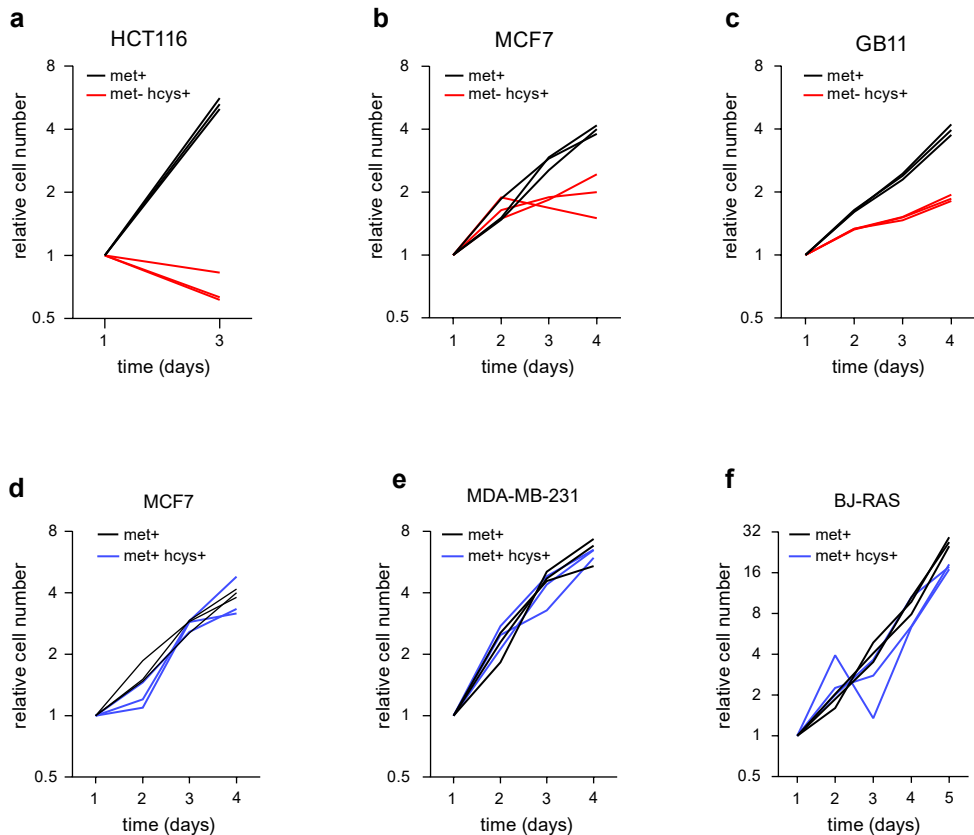

Supplement: Supplementary file 1 — Supplementary Material 1: Figure 1. Methionine dependence in tumor-derived cancer cells. a–c, Growth curves for colon cancer cells, breast cancer cells, lung cancer cellsand glioblastoma cellsin methionine-containingmedium and methionine-free, homocysteine-containing medium. d–f, Growth curves for MCF7 cells, breast cancer cellsand BJ cells transformed with the SV40 Large-T antigen and oncogenic HRASV12, in met+ medium or mediun containing both methionine and homocysteine. Cell numbers relative to day 1 from three independent time course experiments are shown. [file 40170_2025_405_MOESM1_ESM.pdf]

**Supplementary Figure 2**

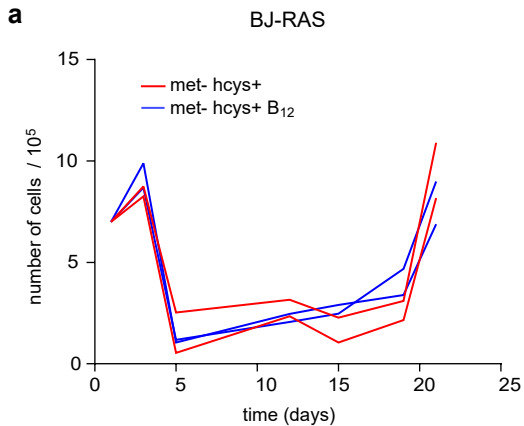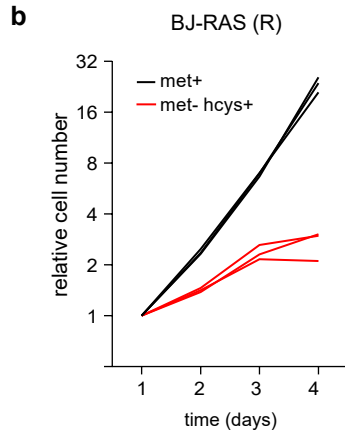

Supplement: Supplementary file 2 — Supplementary Material 2: Figure 2. Reversion of methionine dependence in RAS-transformed cells. a, Long-term growth curves of BJ cells transformed with the SV40 Large-T antigen and oncogenic HRASV12in homocysteine-containing medium with either 0.003 µMor 1.5 µM vitamin B12. Cell numbers in two independent cultures are shown. b, Growth curves of revertant cells BJ-RASobtained from long term cultures, in methionine-containingmedium and methionine-free, homocysteine-containing medium. Cell numbers relative to day 1 from three independent cultures are shown. [file 40170_2025_405_MOESM2_ESM.pdf]

Supplementary Figure 3

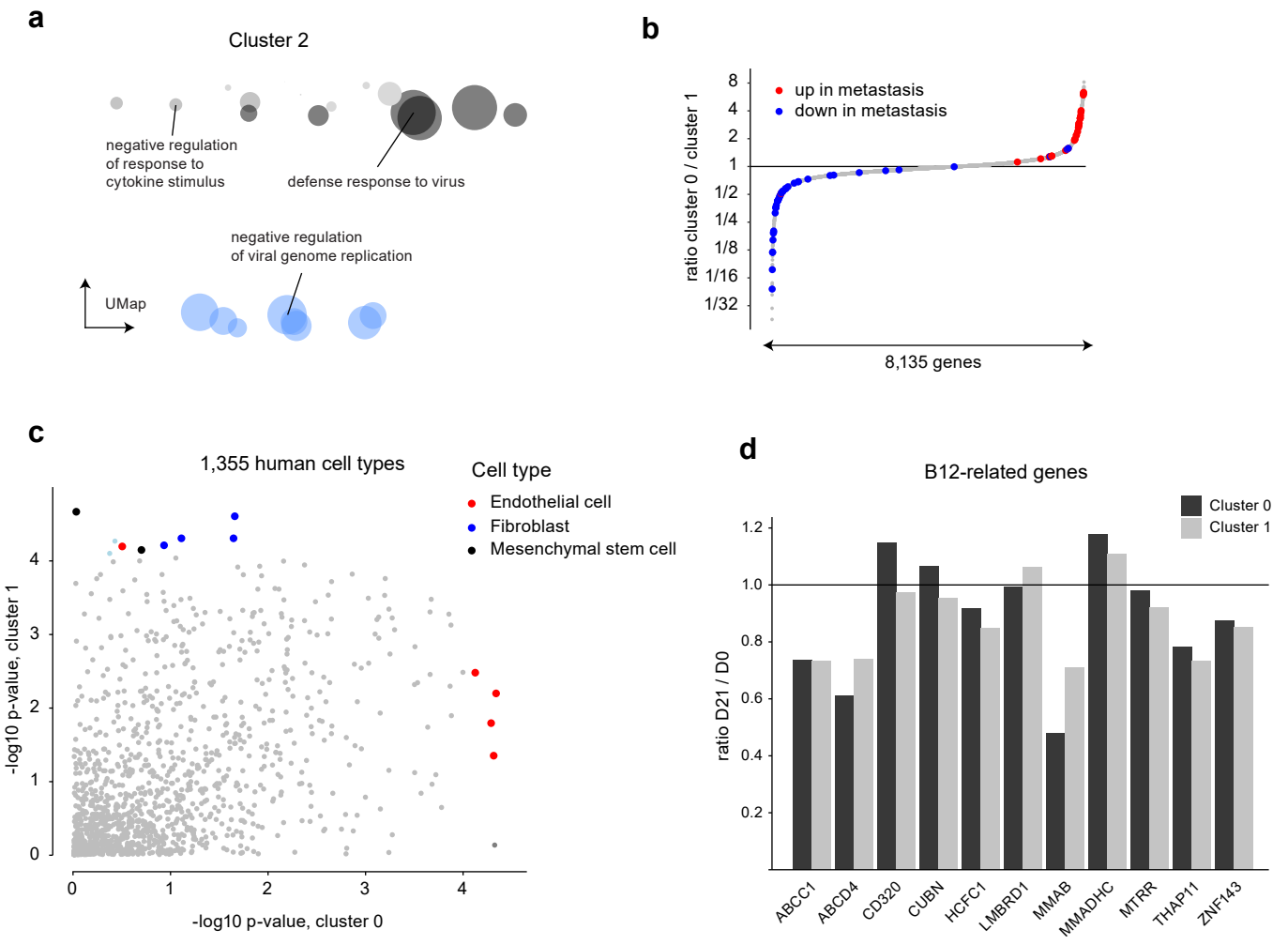

Supplement: Supplementary file 3 — Supplementary Material 3: Figure 3. Gene expression patterns in parental and revertant cells. a, Non-redundant gene ontologypathways enriched in cluster 2, visualized as UMap projection of semantic similarity. Size of circles represent pathway over-representation score. b, S-plot of metastatic signature versus ratio of gene expression in cluster 0 over cluster 1. c, p-values for expression signature match against human 1,355 cell types in cluster 0 and cluster 1. Selected cell types are highlighted. d, Ratio of gene expression levels in D21 over D0 cells for selected genes involved in B12 transport and metabolism. [file 40170_2025_405_MOESM3_ESM.pdf]

# Supplementary Figure 4

**a**

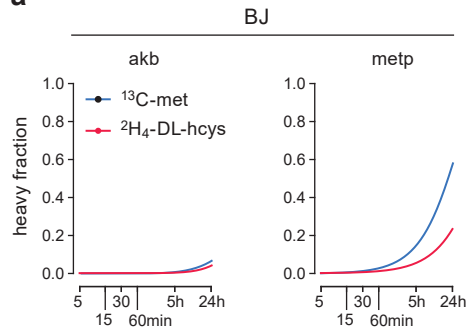

**b**

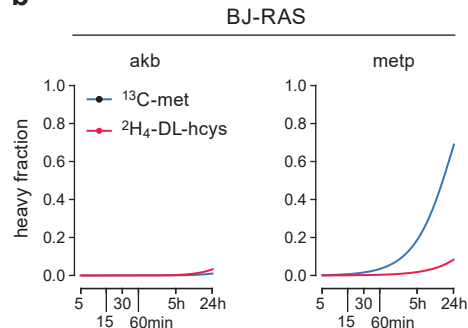

**c**

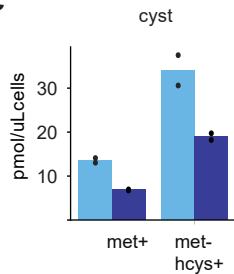

**d**

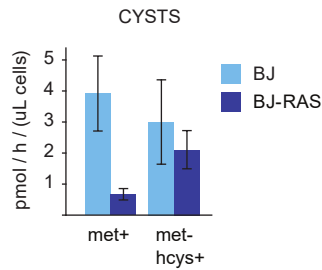

Supplement: Supplementary file 4 — Supplementary Material 4: Figure 4. Metabolic flux analysis in methionine-dependent and -independent cells. a-b, Model predictions for time-course isotope labeling for non-measured metabolites alpha-ketoburytateand protein-bound methioninein BJand BJ-RAScells, in U-13C-methioninemedium and methionine-free medium containing 2H4-DL-homocysteine, at indicated time points. c, Intracellular concentrations of cystathioninein BJ and BJ-RAS cells, in methionine-containingmedium and methionine-free, homocysteine-containing medium. d, Estimated flux through the CYSTS reaction in BJ and BJ-RAS cells, in indicated media. [file 40170_2025_405_MOESM4_ESM.pdf]

# Supplementary Figure 5

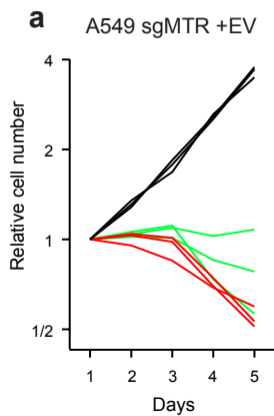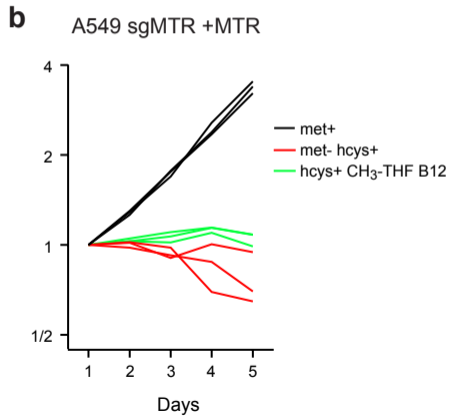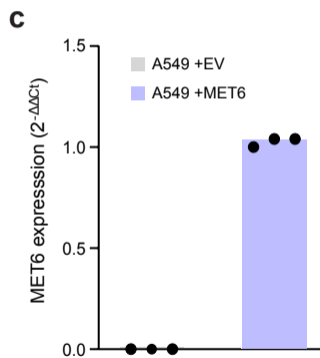

Supplement: Supplementary file 5 — Supplementary Material 5: Figure 5. Cancer cell growth rescue and MET6 overexpression. a–b, Growth curves of A549 cells over-expressing empty vectoror MET6, in methionine-containingmedium; methionine-free, homocysteine-containing medium; and in met–hcys+ medium supplemented with CH3-THF and vitamin B12. c, qPCR of MET6 expression in A549 +EV and A549 +MET6 cels. Data is presented as 2-DDCtfrom 3 replicates. [file 40170_2025_405_MOESM5_ESM.pdf]
